# Bet-hedging in bacteriocin producing *Escherichia coli* populations: the single cell perspective

**DOI:** 10.1038/srep42068

**Published:** 2017-02-06

**Authors:** Bihter Bayramoglu, David Toubiana, Simon van Vliet, R. Fredrik Inglis, Nadav Shnerb, Osnat Gillor

**Affiliations:** 1 Zuckerberg Institute for Water Research, Blaustein Institutes for Desert Research, Ben Gurion University of the Negev, Midreshet Ben Gurion 84990, Israel; 2 Department of Environmental Microbiology, Swiss Federal Institute of Aquatic Science and Technology (Eawag), Ueberlandstrasse 133, 8600 Dübendorf, Switzerland; 3 Department of Environmental Systems Sciences, ETH Zurich, Universitätsstrasse 16, 8092 Zürich, Switzerland; 4 Department of Biology, Washington University in St. Louis, One Brookings Drive, St. Louis, MO 63130, USA; 5 Department of Physics, Bar Ilan University, Ramat Gan 52990, Israel

## Abstract

Production of public goods in biological systems is often a collaborative effort that may be detrimental to the producers. It is therefore sustainable only if a small fraction of the population shoulders the cost while the majority reap the benefits. We modelled this scenario using *Escherichia coli* populations producing colicins, an antibiotic that kills producer cells’ close relatives. Colicin expression is a costly trait, and it has been proposed that only a small fraction of the population actively expresses the antibiotic. Colicinogenic populations were followed at the single-cell level using time-lapse microscopy, and showed two distinct, albeit dynamic, subpopulations: the majority silenced colicin expression, while a small fraction of elongated, slow-growing cells formed colicin-expressing hotspots, placing a significant burden on expressers. Moreover, monitoring lineages of individual colicinogenic cells showed stochastic switching between expressers and non-expressers. Hence, colicin expressers may be engaged in risk-reducing strategies—or bet-hedging—as they balance the cost of colicin production with the need to repel competitors. To test the bet-hedging strategy in colicin-mediated interactions, competitions between colicin-sensitive and producer cells were simulated using a numerical model, demonstrating a finely balanced expression range that is essential to sustaining the colicinogenic population.

Bet-hedging is a strategy that facilitates bacterial survival under fluctuating conditions by switching between phenotypes stochastically rather than in response to environmental cues^1^,^2^. This strategy has been shown to benefit various bacterial populations, such as obligate commensals that, under certain conditions, can turn into human pathogens when they need to avoid recognition by the host’s immune system^3^,^4^, persister cells that switch between growing and non-growing states to avoid the toxic effects of antibiotics^3^,^4^, or soil bacteria that switch between vegetative and dormant states to cope with fluctuating environments^5^. Here, bet-hedging is suggested as the favoured strategy for *Escherichia coli* populations that defend resources and space by producing bacteriocins.

Bacteriocins are the bacterial weapon of choice against close relatives that utilize the same resources and space. Colicins—bacteriocins produced by *E. coli*—are large proteins that kill their competitors by either pore formation in their targets’ membranes or nucleic acid degradation^6^. They are encoded on plasmids in operons that typically contain three colicin-related genes: a toxin-encoding gene, an immunity gene that protects the producing cell, and a lysis gene. Due to lack of a dedicated release system^6^,^7^,^8^, the lysis protein lyses the producing cell to release colicin, and thus colicin expression ultimately results in death to the producing cell while benefiting its clone mates. This strategy can be considered an altruistic trait^9^. Alternatively, the expression of bacteriocins produced by *Pseudomonas aeruginosa*, which share numerous features with colicins, has been suggested to constitute spiteful behaviour as release of the bacteriocin harms the producer while killing the sensitive target cells^10^,^11^. Here, an alternative strategy for bacteriocinogenic populations is proposed: they hedge their bets by phenotypically alternating between expressers and non-expressers of bacteriocins.

It is assumed that colicins confer a competitive advantage to the producing strains, even though the fitness of the colicinogenic cells is compromised probably due to colicin synthesis and the lethality of production^12^,^13^,^14^. The high cost imposed by colicin production suggests that extensive measures are required to avoid it by tightly controlling expression^15^,^16^. Another measure suggested to compensate for the cost associated with production colicins is the ‘division of labor’ as only a very small fraction of the population express colicin, whereas most of the cells silence expression, suggesting phenotypic heterogeneity^17^. Studies evaluating the number of producers of various colicin types revealed that, under specific environmental conditions^18^, 0.5–3% of planktonic *E. coli* populations and 7–9% of sessile populations produce colicins^19^,^20^,^21^. Stochastic expression of colicins in monoclonal populations has been shown to be regulated by the SOS response system^22^,^23^, suggesting that random mutations induce the DNA-repair system, which triggers colicin expression. Moreover, nutrient-responsive regulators have been shown to adjust the production and/or release of different colicins: Ib^24^, K^15^, E7^25^ and E2^18^.

Although the heterogeneous expression of colicins has been studied in detail, the characteristics of the genetically identical but phenotypically different subpopulations are unknown. We studied the dynamics of colicin expression at the single-cell level by tracking colicinogenic populations as they proliferate from a single cell. Using time-lapse microscopy, three strains, carrying colicins A, E2 and E7, respectively, were followed. Colicin-expressing cells carried a fluorescent reporter for colicin production, thus allowing segregation to distinguish between two subpopulations: the majority with silenced expression, and the rare minority of expressers. The colicin expressers were significantly different from their non-expressing clone mates, probably because their DNA was damaged, thus inducing the SOS response system^22^,^23^. We predicted that these expressers persist until enough lysis proteins accumulate to lyse the producer cell, releasing colicins into the media. However, we hypothesised that different colicinogenic strains would diverge in their expression patterns in accordance with the differences in their regulatory elements^16^,^26^,^27^,^28^ and modes of action^6^.

## Results

### Colicins exhibit heterogeneity in sessile populations

Expression of three types of colicin—A, E2 and E7—was followed over time in sessile cultures using time-lapse microscopy. Colicin A was chosen as it kills by forming pores in its target’s membrane^6^ and its expression has been found in ~0.6% of a given population^22^. Colicins E2 and E7 are both nucleases: colicin E2 has been shown to be expressed by ~6% of a given population^28^, whereas colicin E7 was expressed by ~2%^22^,^28^. Thus, colicin-mediated heterogeneity could be tested in three populations of high, medium and low expressers (corresponding to colicins E2, E7 and A, respectively).

The colicinogenic strains were genetically identical except in their colicin operons, cloned in a medium-copy-number plasmid (pBR322), and the respective promoter regulating a low-copy-number reporter vector (pUA66). Isogenic strains lacking the colicin operon but hosting a reporter vector regulated by colicin E2 promoter were used as controls—these were chosen for their classical structure^16^. The fluorescent reporter allowed us to differentiate between cells that silence colicin expression and those that express colicins.

We estimated the average expression at five time points of each colicin used in this study, and compared it with expression levels reported in previous studies. The expression results corresponded with reported values and were observed in a subset of the populations, as reported previously^22^,^28^. In addition, expression levels differed among strains; while colicin E7 was expressed in only 1 ± 0.2% of the population, the auto-inducer strain E2^18^ was expressed in 8 ± 1.5% and colicin A in 2 ± 0.4% of the population. However, expression of the three colicinogenic populations was not stable over time, even though the number of expressers was roughly constant (Supplementary Videos S1, S2 and S3). Individual cells switched between expressing and non-expressing states, forming hotspots within the sessile populations (Supplementary Videos S1, S2 and S3). These hotspots were dynamic, as colicin-expression levels changed over time, increasing or decreasing with cell division. The videos show that daughter cells of colicin expressers may switch to being non-expressers while non-expressers may start expressing over time (Supplementary Videos S1, S2 and S3). Moreover, colicins seemed to alter their host cell morphology, as expressing cells were elongated and their doubling time was delayed. To further explore colicin-mediated changes in the cells’ morphology, we carefully followed expressers and non-expressers by analysing the time-lapse microscopy images.

### Cell length in the colicinogenic strains

**Figure 1:**
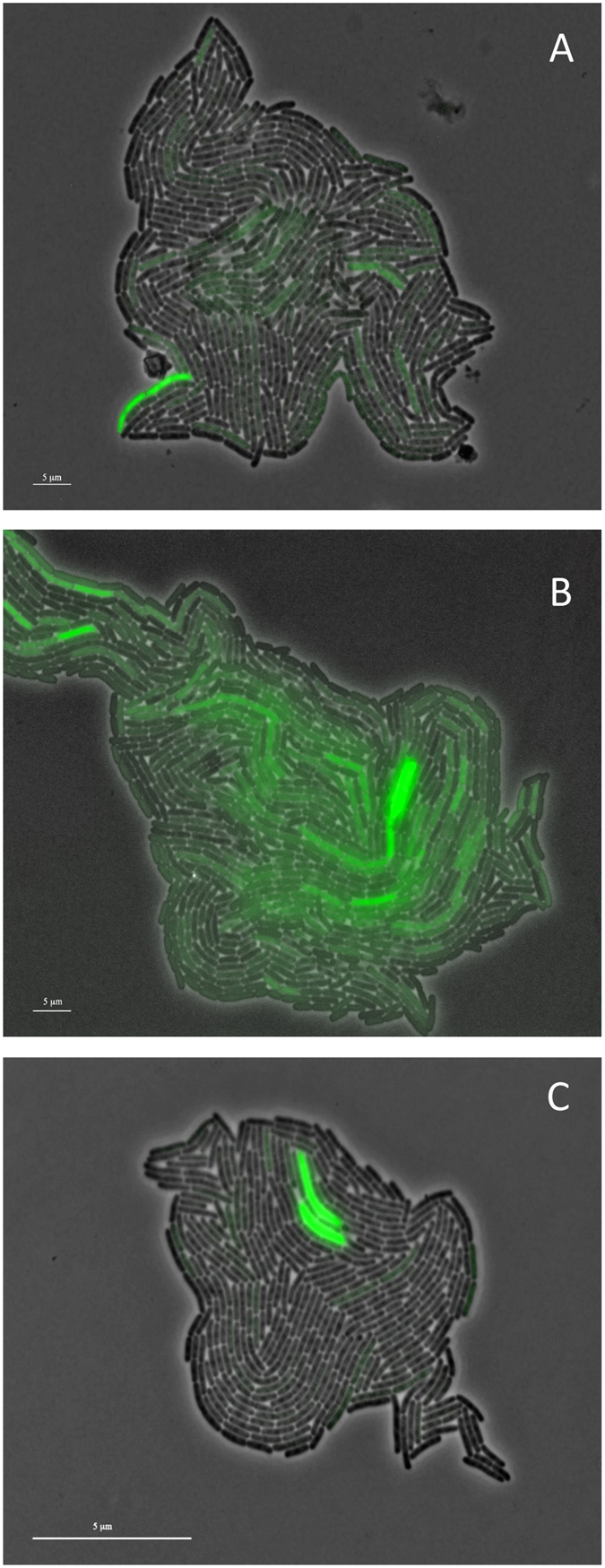
Fluorescence images showing heterogeneity in colicin expression. Expressions of colicin A (**A**), colicin E2 (**B**) and colicin E7 (**C**) are represented by green fluorescence.

**Figure 2:**
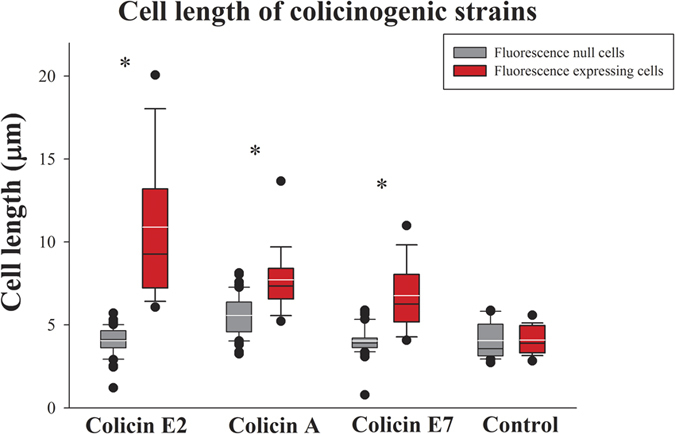
Cell length of colicinogenic strains and strain lacking colicin-encoding genes. The lengths of colicin expressers (red box) and non-expressers (gray box) were compared for colicins A, E2 and E7 (n = 50) and the control population (n = 20), depicted by fluorescence emission. Error bars refer to the 10/90^th^ percentiles of the tested data and whiskers to 5/95^th^ percentile. White line indicates mean and black line indicates median. The asterisk (*) points to significant differences between the lengths of fluorescent cells and cells that did not emit fluorescence (*P* < 0.001 by Student’s t-test).

Figure 1 shows representative images of cells expressing colicins A (Fig. 1A), E2 (Fig. 1B) and E7 (Fig. 1C). The lengths of the colicin-expresser and non-expresser cells were compared in induced and non-induced populations, and in the control population lacking the colicin-encoding plasmid. Cells of the colicin-expresser subpopulation were significantly longer (*P* < 0.001) (Supplementary Table S1) than their clone mates that did not express colicins or the isogenic strain that did not carry a colicin operon (Supplementary Video S4), indicating colicin-mediated heterogeneity (Fig. 2).

To further explore the link between colicin expression and the imposed changes in cell morphology, the colicinogenic strains were induced with a classical colicin trigger, the DNA-damaging agent mitomycin C^29^. Induced colicin-expressing cells showed the expected increase in colicin expression compared to non-induced colicin expressers (Supplementary Videos S5, S6 and S7) and the changes in cell length were enhanced (Supplementary Fig. S1). Induced colicin A- and E7-expressing cells were significantly longer than the non-induced expressing cells (*P* < 0.01), but colicin E2 expressers did not differ significantly upon induction (Supplementary Fig. S1). Similarly, non-expressing cells did not differ in their cell length, regardless of induction (Supplementary Fig. S1).

### Doubling time in colicinogenic strains

**Figure 3:**
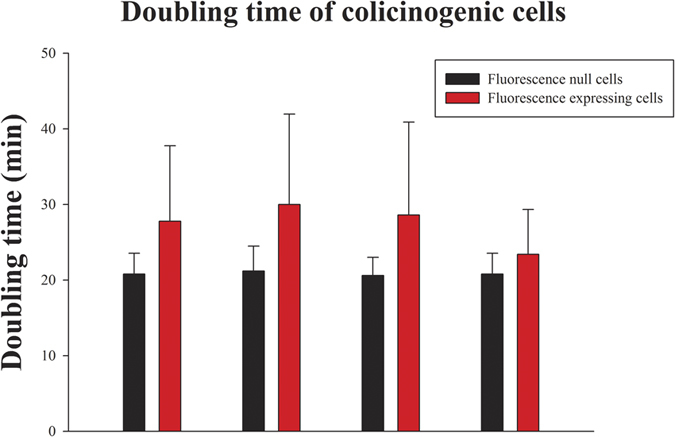
Cell doubling times for colicinogenic strains and strain lacking colicin-encoding genes. Bars represent the average cell doubling time and standard deviation for expressers (red bar) and non-expressers (black bar) of colicins A, E2 and E7 (n = 50) and the control population (n = 20).

To estimate the changes in fitness between colicin expresser and non-expresser subpopulations, their doubling time was evaluated (Fig. 3) as well as the growth rate (Supplementary Table S2). The slight increase in colicin expressing cells’ doubling time was insignificant (Supplementary Table S1). We could not detect any trend differentiating the colicinogenic strains from the controls as doubling time increased similarly in all of the strains (*P* = 0.62). However, induction of colicin expression significantly (*P* < 0.01) delayed cell division in all colicinogenic strains (Supplementary Fig. S2).

### Cell lineages and colicin-expression trajectories

**Figure 4:**
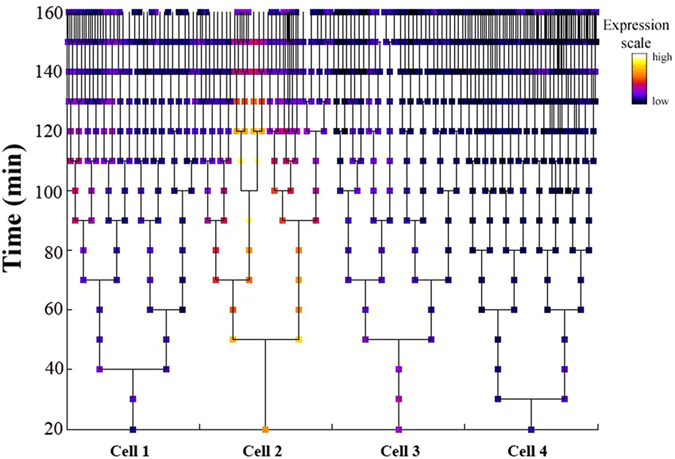
Representative cell lineage of strain expressing colicin E2. Colicin expression was monitored over time (y axis) along the lineages of four cells. Expression is depicted by colour: dark cells are non-expressers while lighter cells, due to higher fluorescence emission, mark colicin expression.

Colicin expression was followed over time by tracking the cell trajectories along their respective lineages. Expression was depicted by colour: dark cells were non-expressers while the lighter cells, presenting higher fluorescence emission, marked colicin expression. As previously shown^22^,^28^, except for a small subset, most of the colicinogenic cells did not express colicins. The expression of colicin E2 was higher (Fig. 4) than that of colicins A (Supplementary Fig. S3) and E7 (Supplementary Fig. S4). For technical reasons, it was not possible to follow the cells beyond the 20^th^ time point and in some strains (colicin E2 expressers and the control strain), analysis was limited to the 16^th^ time point. However, the lineages suggested stochastic switching between phenotypic states, i.e., between colicin expression and silencing. Following single cells suggested that they can gain or lose the ability to express colicins over time. Figure 4 shows a lineage that begins with four cells, one of them fluorescent. However, not all of its daughter cells expressed colicins; in fact, some switched to non-expressing cells after the 12^th^ time point. On the other hand, daughter cells of non-expressers switched and fluoresced after the 9^th^ time point (Fig. 4, lineage on left-hand side). The lineages of colicin A and E7 expressers showed similar trajectories (Supplementary Figs S3 and S4, respectively), stochastically switching between colicin expression and non-expression. Low expression was detected in the control lineage, switching on at the 10^th^ time point, and then this trait was silenced. However, the morphology of the fluorescing cells was not altered (Supplementary Fig. S5).

Interestingly, the lineages of colicin E7-expressing cells suggested that colicin expression was initiated after 20^th^ time point and that they were then lysed at 22^nd^ time point (Supplementary Fig. S4). We note that lysis events were rare or not observed in the other colicinogenic strains (E2 and A). This could be attributed to the M9-glucose media used for growing the cells in this study, as it has been shown that sugars drastically decrease the lysis rate of colicin-producing cells whereas amino acids and small peptides enhance the production and release of colicins^18^.

### Numerical model

**Figure 5:**
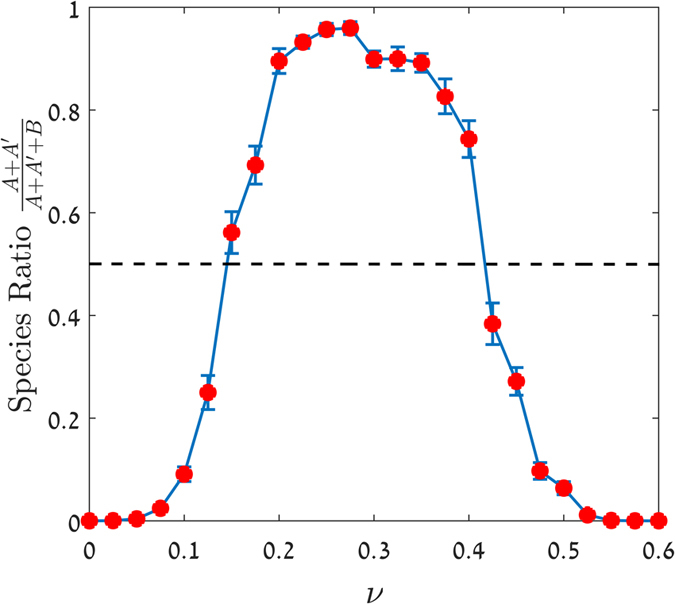
Colicin-mediated competition between producer and sensitive strains. The population ratio A/(A + B) is shown after 400 generations for a community comprised of colicin producers (**A**) and colicin-sensitive strains (**B**). The population ratio is plotted against the probability (ν) that strain A will produce colicin. For all strains, μ = 1 and the doubling rates are λ_A_ = 4, λ_A’_ = 2.5 and λ_B_ = 5. A’ cells can lyse, thereby killing neighbouring B strain cells, with a probability of 0.5; when the A’ cell does not lyse, it either dies or divides. The phenotype change from A to A’ or vice versa occurs with probability ν. The simulation was performed on a 50 × 50 square lattice with periodic boundary conditions, each point representing an average of 20 samples with the same ν value, each initiated with random initial conditions. Error bars represent one standard deviation.

**Figure 6:**
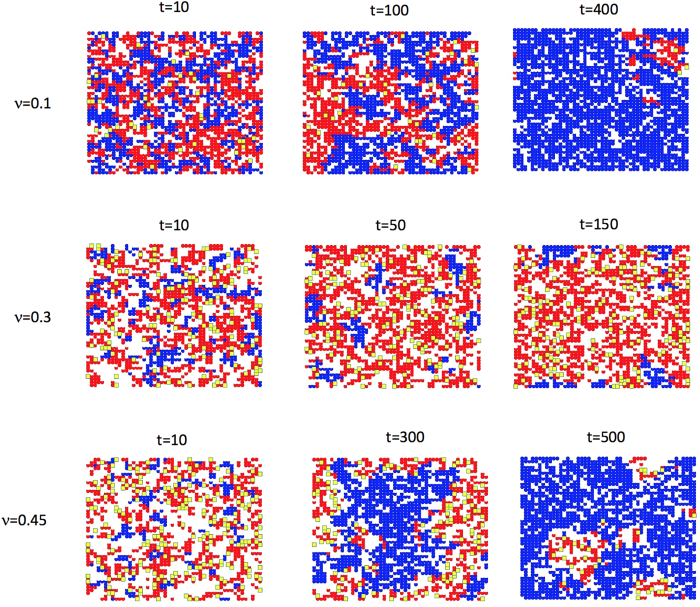
Snapshots of the spatial configuration of colicin-producer (**A**) and colicin-sensitive (**B**) strains for various time points and phenotype diversity probability ν. We simulate competition between population B (blue), population A (red) which is comprised of colicin carriers, and subpopulation A’ (yellow) that actively produces colicins. Note that the timescales needed for fixation differ. When ν = 0.1, the number of A’ cells is small and population B dominates since the doubling rate of A (λ_A_) is smaller than that of B (λ_B_). When ν = 0.3, the number of active colicin producers (A’) is large enough for A to dominate the lattice. When ν = 0.45, there are many active colicin producers (A’) that initially dominate the lattice but then excess A’ cells lead to clustering and inefficient use of the costly colicin, thus allowing B cells to take over.

Our results portrayed novel phenotypes of colicin-producing populations that had not been considered in previous modelling of colicin-mediated interactions^1^. To test the interactions between colicin producers and their adversaries, while considering the observed phenotypic changes, a numerical model was used in which a colicin-producing population (strain A) competes with a colicin-free population (strain B). Pursuant to published studies^14^ and the experimental results presented here, the strains were assigned the following traits: (i) the fitness of strain A is lower than the fitness of strain B^14^; (ii) a subset of population A produces colicin, thus forming subpopulation A’ (Fig. 1); (iii) upon binary fission, the daughter cells of A’ can show either the A or A’ phenotype (Fig. 4); (iv) only a subset of the A’ subpopulation lyses, releasing colicins and killing the competitors (i.e., population B). The simulated competition between strains A and B was followed over time and under different conditions (Figs 5 and 6).

The appearance of the colicin-expresser phenotype (subpopulation A’) increases the overall fitness of population A, since A’ can kill the competitor and thus free up resources. However, the cost of production can impair the overall fitness of the population. Therefore, if the proportion of A’ cells in the A population is too high, the outcome is negative (Fig. 5), since the overall reproduction rate (λ) cannot match the death rate (μ). Moreover, because reproduction is local^30^, the expresser cells (A’) will aggregate and will not be evenly dispersed in population A, limiting the efficiency and benefit of lysis and colicin release. Accordingly, the population ratio is a unimodal function of the probability of producing colicin (ν). Figure 5 shows that when population A’ is in low abundance, meaning that the chance to initiate colicin production is small, then population B will dominate because there are not enough colicin producers to kill the B cells. Large ν values will also lead to dominance of the colicin-sensitive cells (B), as the combination of lysis and the lower doubling time of subpopulation A’ will hinder their ability to compete. Population A will only dominate in a narrow range of ν that achieves the right balance between the cost of colicin production and the abundance of competitors.

Snapshots of the scenarios depicted in Fig. 5 are detailed in Fig. 6: when the values of ν are very low, B cells outcompete A cells (Fig. 6, top row); at intermediate ν values, the competition is resolved by the colicin-producer cells (A) taking over the lattice (Fig. 6, middle row). However, when ν is very high, the fitness of population A decreases because too many cells are being lysed within the community, thereby reducing the fitness of strain A and driving strain B to dominate the lattice (Fig. 6, bottom row).

## Discussion

The prevailing model of colicin production asserts that colicinogenic populations are heterogeneous, as a small subset expresses large amounts of colicin, which increase during induction^17^,^19^,^20^,^22^,^23^. Phenotypic heterogeneity disperses expression costs among members of the clonal population and has been reported in bacteriocins produced by *Bacillus subtilis*^31^, *Streptococcus mutans*^32^ and *Streptococcus pneumoniae*^33^. Nevertheless, colicin expression is considered lethal to the producing cell due to its association with the lysis protein responsible for releasing the toxin^6^. The producer cell’s fitness is lowered under natural conditions due to a substantial cost of carriage^14^. Our experiments suggest that in early stages of development (up to 200 cells), the cost associated with colicin expression entails marked changes in the cell’s morphology and growth patterns.

Colicinogenic populations were tracked over time starting from a single cell, by following its proliferation and colicin expression as the population expanded (Supplementary Videos S1, S2 and S3). Strains differed only in their colicin-encoding operons; all other molecular features were identical. Three colicin-producing strains, expressing colicins E7 (low rate), A (medium rate) and E2 (high rate) were compared. The colicinogenic populations diversified into two distinct, albeit dynamic subpopulations: one containing most of the cells, with silenced colicin expression, and the other containing a small number of phenotypically unique cells forming randomly distributed hotspots of colicin expression (Supplementary Videos S1, S2 and S3). It should be noted that colicin production is costly and thus tightly regulated^15^,^23^,^34^. In a natural environment, stochastic SOS stress has been shown to induce colicin expression in a small subset of the colicinogenic population^22^. Here, colicin expression was shown to tax the subpopulation by being associated with cell elongation (Fig. 2) and delayed fission (Fig. 3).

*E. coli* populations have been shown to elongate and form bulges when exposed to antibiotic stress^35^,^36^, or when their DNA is damaged and the SOS system is triggered^37^,^38^. During the SOS response, separation is blocked and cell division is inhibited leading to elongation and sometimes filamentation_39,40_. Here, time-lapse microscopy showed that stochastic phenotype switching from non-expressers to colicin expressers entails significant elongation (Fig. 2). We suggest that the stochastically dispersed colicin-expressing *E. coli* cells exhibit elongation (Fig. 2) in response to stress inflicted by DNA damage^41^. Moreover, elongation was not observed in the colicin-free strain (Fig. 2), suggesting that colicin expression enhances DNA damage regardless of the mode of colicin activity.

Under SOS stress, the colicin-expressing cells seldom lysed, as their daughter cells shed the burden of colicin expression by switching their phenotype spontaneously and reversibly to join the majority—the colicin-silenced cells (Fig. 4). Two types of phenotypic switching have been described: responsive switching, which occurs in response to external cues sensed by the bacteria, and spontaneous stochastic switching, which occurs without any direct sensing of the environment^42^. Although colicin production is considered a first line of defence for *E. coli* against competitors, it is not induced in response to said competitors (in contrast to the Gram-positive bacteriocins)^21^ but by the producer cell’s regulatory system^6^. The phenotypic switch detected here (Fig. 4) was independent of external induction and therefore, we propose that switching between colicin expressers and non-expressers is spontaneous and not responsive. A previous modelling study showed that stochastic switching can be favoured over sensing when the environment changes infrequently^42^.

In the never-ending race for resources and space, a given colicinogenic population needs to be constantly on the lookout, ready to deter competitors. On the other hand, the cost of colicin production is substantial and impairs the population’s fitness. The results of this study suggest that the colicinogenic populations are hedging their bets by stochastically switching between phenotypic states, thus alternating between paying the cost of colicin expression and succumbing to the external threat of competition. To the best of our knowledge, bacteriocin production has never been suggested to be a bet-hedging strategy, perhaps because the phenotypic switching reported here (Supplementary Videos S1 and Fig. 4) had not been demonstrated.

To explore the bet-hedging strategy in the context of natural selection, a numerical model that simulated competition between a colicin-sensitive and producer strain was used. The colicin producers were attributed the ability to stochastically switch back and forth between colicin-silencing and expressing states, where only a subset of the expressers released the colicin by lysis. The model demonstrated a finely balanced range of sustainable probabilities for the colicin-producing cells (Fig. 5). Above or below this range, the cells were outcompeted by their adversaries but within it, they benefited from colicin production, and were unharmed by the fitness cost as they successfully competed and eventually dominated the community.

## Methods

### Bacterial strains and plasmids

All experiments were performed with *E. coli* strain MG1655. Three colicinogenic strains were chosen as representative expressers of colicins at high, medium and low levels^22^,^28^. Three types of colicin were studied: colicin A to represent colicins that kill by forming pores in their target’s membrane^6^; colicins E2 and E7 to represent colicins that kill by non-specific DNA cleavage^6^, with enhanced expression of the former compared to latter as it is an autoinducer^28^. Supplementary Table S3 lists the strains and plasmids used in this study.

To explore the effect of colicins on the heterogeneity of the *E. coli* population, a set-up that ensured an identical plasmid backbone for all strains was used such that they differed only in their colicinogenic traits. Each of the colicinogenic strains used in this study hosted two plasmids (Supplementary Table S3): pBR322 containing the colicin operon (pBR-ColA, pBR-ColE2, or pBR-ColE7) and the reporter plasmid pUA66 regulated by the respective colicin’s promoter (pUA66-ColA, pUA66-ColE2, or pUA66-ColE7, respectively). In addition, a strain that hosted the reporter plasmid regulated by the colicin E2 promoter was used as a control for colicin expression and putative effect on cell morphology and behaviour. The plasmid chosen to host the colicin operon (pBR322) was based on the plasmid encoding colicin E1 and was of medium copy number^43^. The reporter plasmid pUA66 was of low copy number and included *gfpmut2* with a strong ribosome-binding site as the reporter gene^44^. We note that a similar reporter vector, based on colicin D promoter was previously demonstrated to be a highly sensitive reporter to various genotoxic agents^45^.

### Plasmid construction

The promoter regions of colicins A, E2 and E7 were amplified as previously described^16^. Primer sets were designed with XhoI and BamHI restriction sites (Supplementary Table S4). The resulting amplicons and pUA66 vector were digested with XhoI and BamHI restriction enzymes and fused to form plasmids pUA66-ColA, pUA66-ColE2 and pUA66-ColE7.

All plasmids were purified using AccuPrep Plasmid Extraction Kit (BioNeer, Seoul, South Korea) and transformed into *E. coli* strain MG1655 in pairs (pBR-ColA and pUA66-ColA; pBR-ColE2 and pUA66-ColE2; pBR-ColE7 and pUA66-E7; pBR322 and pUA66-ColE2) resulting in four strains. Transformants were selected on the basis of antibiotic resistance and their identities were confirmed by PCR.

### Strain validation

To establish the link between fluorescence and colicin expression, a representative strain harboring the plasmids pBR322-ColE2 and pUA66-ColE2 (Supplementary Table S3) was monitored. The mRNA transcribing the genes encoding green fluorescent protein and the colicin E2 protein were monitored using real time quantitative PCR (qPCR). Our results suggest strong inter-gene correlation between the expression of the colicin activity protein and the GFPmut2 (r = 0.95; Supplementary Fig. S7). For more details please see supplementary information. The high Pearson’s coefficient of correlation indicates that the expression pattern of colicin E2 is similar to the reporter protein thus justifying the use of fluorescence as colicin proxy.

### Growth conditions

M9 minimal salt medium (Sigma, Rehovot, Israel) supplemented with 0.4% glucose was prepared according to the manufacturer’s instructions and supplemented with ampicillin (100 mg L^−1^, Sigma) and kanamycin (50 mg L^−1^, Sigma) as required. Suspended cultures were grown in an incubator at 37 ± 0.5 °C with shaking at 200 rpm and were used as inoculum for the sessile cultures. Sessile cultures were grown on M9 agar pads supplemented with the same concentrations of glucose, ampicillin and kanamycin at the same temperature.

### Microscopy

Agar pads of M9 minimal medium supplemented with ampicillin and kanamycin were prepared as previously described^46^. For induction experiments, the medium was supplemented with a sublethal dose of Mitomycin C at a final concentration of 50 ng mL^−1^. The pads were inoculated with 1:500 dilutions of an overnight culture of the colicinogenic cells. The agar pads were closed with a coverslip and sealed with vacuum grease. Under these conditions, cells can grow exponentially as a monolayer for many generations^47^. The slide was mounted onto an automated Olympus IX81 microscope with UPLFLN100xO2PH/1.3 phase-contrast oil lens (Olympus, Volketswil, Switzerland). The cells were incubated at 37 °C and images were recorded every 10 min. Each experiment was performed in biological duplicates, and 10 technical replicates were performed per agar pad.

### Image analysis

Images were analysed using Fiji, an open source image-processing package based on ImageJ (available at fiji.sc/Fiji). The resulting image sequences were further analysed with the Matlab-based script package ‘Schnitzcell’^48^ with custom modifications. Schnitzcell was used to segment the images, and identify and track cells through consecutive images. Data were then further processed and plots were generated using SigmaPlot 12.5 (Systat Software Inc., San Jose, CA). Cell lineage data were extracted and generated with customised Matlab scripts.

To distinguish expressers and non-expressers we used a five-fold threshold that refers to the least fluorescent cell in any given image analyzed. To characterize the morphology of colicin expressers and non-expressers, cell lengths of the 50 most fluorescent and 50 least fluorescent cells, as defined by Schnitzcell^48^, were used. As a control, cell lengths of non-colicinogenic populations hosting pUA66-ColE2 were recorded, differentiating between fluorescent and non-fluorescent cells.

To calculate the doubling time of colicin expressers and non-expressers, cells were randomly selected at various time points of the experiment based on the data provided by the Schintzcells software. During the experiment images were taken every 10 min, then the software analyzed these images by following individual cells (fluorescent and non-fluorescent) and measuring the time (in minutes) between consecutive divisions. Doubling times estimated using reconstructed images of the respective images were then summarized and averaged for each group.

In mitomycin C induced populations, all the cells had higher fluorescence than uninduced cells, therefore we did not define any of these cells as ‘non-expresser’ but as ‘lower expressers’ setting a threshold of less than 10% of the population defined by relative intensity of the detected fluorescence emission. The differences in fluorescence emission were used to define the two groups of lower and higher expressers and the doubling time estimations were performed as described above.

To visualize the individual cell linages over time we colored the cells by scaling each according to intensities gradient ranging from minimal (=0) and maximal value (=1). These values used are the mean background corrected fluorscence intensities of each cell. Each cell was then assigned a color ranging from black (0) to white (1) and was used to construct the population linage.

### Statistical analysis

Statistical analysis was performed using SigmaPlot 12.5. Doubling time and length of colicin expresser and non-expresser cells under induced and non-induced conditions were analysed and compared by one-way ANOVA. When ANOVA indicated significance, Tukey’s test for pairwise comparison was used for *post hoc* analyses.

### Numerical modelling

The spatially explicit dynamics of the colicinogenic population was modelled by using the contact process^49^. For a single population, the model is characterized by two rates: the death rate μ and the reproduction rate λ. During each elementary time step, one individual is picked at random, which then dies with probability μ/(λ + μ) or tries to reproduce with probability λ/(λ + μ). Only one individual is allowed at each site, so after binary fission, the daughter cell chooses a neighbouring site at random, capturing this target site if it is empty or dying if it is occupied.

To model the competition between the colicinogenic (population A) and colicin-free (population B) strains, one can consider a spatial system with both A and B strains, and with a set of death rates μ_A_ and μ_B_ and doubling rates λ_A_ and λ_B_. Again, only one individual is allowed in any one site, so the daughter cell may capture a site only if it is empty (it cannot enter a site occupied by the other strain). Without loss of generality, one might take the death rates to be the same, μ_A_ = μ_B_ = 1, meaning that time is measured in units of generation time. In this case, the strain with the higher reproductive rate will win, i.e., it will invade the regions occupied by the weak strain and will drive it to extinction.

Strain B was assigned higher fitness compared to strain A, thus λ_A_ = 4 and λ_B_ = 5. If colicin production is not mediating the competition, strain B will out-select the weaker strain, A (Supplementary Fig. S6). To increase its fitness under the competition with strain B, strain A may benefit from producing colicin (selectively killing strain B). Thus strain A cells can all produce colicin but only a subset of the population expresses this trait. Upon binary fission, the daughter cell in population A can either produce colicin with probability ν, or silence colicin production with probability 1-ν. The colicin-producing subpopulation was designated A’. This process is “reversible” as upon binary fission, the daughter cell of A’ can either feature the A’ phenotype (colicin producer) with probability 1-ν or the A phenotype (silencing colicin production) with probability ν. In other words, upon division of an A’ cell, the daughter cells will be a producer with probability 1-ν and a non-producer with probability ν, meaning that the probability that a descendent of a producer will also be a producer after k generations is distributed geometrically and the mean number of generations before memory loss is 1/ν. This leads to the spatial aggregation of producers observed in the simulations.

Due to the extra cost of synthesizing the colicin and lysis proteins, the doubling rate of subpopulation A’, λ_A_’, is lower than λ_A_. However, when lysis occurs, the colicin produced by a cell of subpopulation A’ is released, killing a strain B cell in its vicinity. In our model, the colicin producer cell vicinity is defined as the eight sites surrounding the lysed A’ cell, i.e., nearest neighbours and second nearest neighbours.

## Additional Information

**How to cite this article**: Bayramoglu, B. *et al*. Bet-hedging in bacteriocin producing *Escherichia coli* populations: the single cell perspective. *Sci. Rep.* **7**, 42068; doi: 10.1038/srep42068 (2017).

**Publisher's note:** Springer Nature remains neutral with regard to jurisdictional claims in published maps and institutional affiliations.

## Supplementary Material

Supplementary Information

Supplementary Video S1

Supplementary Video S2

Supplementary Video S3

Supplementary Video S4

Supplementary Video S5

Supplementary Video S6

Supplementary Video S7
