# Supplementary material for: Bet-hedging in bacteriocin producing Escherichia coli populations: the single cell perspective
Source: Sci Rep. 2017 Feb 6;7:42068. doi: 10.1038/srep42068 (PMC5292716; doi:10.1038/srep42068)
Supplement: Supplementary Information [file srep42068-s1.pdf]

Bet-hedging in bacteriocin-producing *Escherichia coli* populations: the single-cell perspective

Bihter Bayramoglu, David Toubiana, Simon van Vliet, R. Fredrik Inglis, Nadav Shnerb, Osnat Gillor

Supplementary Information

Colicin – fluorescence correlation

To establish the link between fluorescence and colicin expression we monitored gene expression of the green fluorescent protein (*gfp-mut2*) and colicin E2 activity (*ce2a*) encoding genes, with and without induction. To that end, *E. coli* cells harboring pBR322-ColE2 and pUA66-ColE2 plasmids (Table S2) were grown to early exponential phase (OD_{600} of ~0.07) and then divided in two: the control group was cultivated to stationary phase for 5 h (OD_{600} ~ 1). In the treatment group the cells were induce for colicin expression[1] by supplementing them with 50 ng mL^{-1} of the colicin inducing agent Mitomycin C (Sigma) for 5 h (OD_{600} ~ 1). The experiment was terminated by adding glycerol to 20% (v/v), the mixture was placed liquid nitrogen and then stored in -80 °C. Each experiment was repeated at least three times.

The freeze cells were thawed on ice and total RNA was extracted using MasterPure RNA purification kit (Epicentre, Madison, WI) according to the manufacturer's instructions. The total RNA was quantified using Quant-iT™ RiboGreen (Molecular Probes, Eugene, OR) and cDNA was then synthesized from

the RNA using RT-PCR Kit ImProm-II™ Reverse Transcription System (Promega, Madison, WI) according to the manufacturer's instructions.

The qPCR primers of the respective target genes (Table S3) were designed using Geneious [2]. All PCR amplifications were performed in iCycler thermos-cycler equipped with MyiO detection system (Bio-Rad, Hercules, CA) and the data was processed using Bio-Rad CFX Manager 3.0 software (Bio-Rad). The reaction was adjusted to a final volume of 20 µl containing 5 µl cDNA (adjusted to 2.5 ng), 3 µl of molecular grade water (Sigma-Aldrich), 1 µl of each primer (400 nM final concentration) and 10 µl SYBR_Green PCR Master Mix (Thermo Fischer, Waltham, MA). All qPCR reactions were performed in duplicates under the following conditions: 95 ºC for 5 min, followed by 40 cycles of 94 ºC for 30 s, 60 ºC for 30 s and 72 ºC for 30. Melting curve analysis was done using the default settings of the device (BioRad).

To standardize the results we used known amounts of the extracted plasmids pBR322-ColE2 and pUA66-ColE2 (Table S2). Calibration curves utilized the known concentrations of the plasmids plotted against the Ct values. We then used the calibration curves to calculate the copy number of the expressed genes in the samples. Gene expression was standardized to 1 ng RNA. The data set was pairwise compared to determine the Pearson correlation coefficient.

Our results suggest strong inter-gene correlation between the expression of the colicin activity protein and the GFPmut2 (r = 0.95; Figure S7). The high Pearson's coefficient of correlation indicates that this gene pair has very similar overall expression patterns.

Figure legends

Figure S1. Cell length of colicinogenic strains under induced conditions. The length of colicin expressers (red box) and non-expressers (grey box) was compared for colicins A, E2 and E7 (n = 50) depicted by fluorescence emission. Error bars refer to the 10/90th percentiles of the tested data and whiskers to 5/95th percentile. White line indicates mean and black line indicates median. White line indicates mean and black line indicates median. The asterisk (*) points to significant differences between the length of fluorescent cells and cells that did not fluoresce ($P < 0.001$ by Student's t-test).

Figure S2. Cell doubling time of colicinogenic strains under induced conditions. Bars represent the average cell doubling time and standard deviation for expressers (red bar) and non-expressers (black bar) of colicins A, E2 and E7 (n = 50 for E2, n = 20 for A, E7). Asterisk (*) indicates significant differences between the doubling time of fluorescent cells and cells that did not emit fluoresce ($P < 0.001$ by Student's t-test).

Figure S3. Representative cell lineage of strain expressing colicin A. Colicin expression was monitored over time (y axis) along the lineages of two cells. Expression is depicted by colour: dark cells are non-expressers while lighter cells, due to higher fluorescence emission, mark colicin expression.

Figure S4. Representative cell lineage of strain expressing colicin E7. Colicin expression was monitored over time (y axis) along the lineages of one cell.

Expression is depicted by colour: dark cells are non-expressers while lighter cells, due to higher fluorescence emission, mark colicin expression.

Figure S5. Representative cell lineage of control strain lacking the colicin-encoding genes. GFP expression was monitored over time (y axis) along the lineages of one cell. Expression was depicted by color: dark cells are non-expressers while lighter cells mark expression such that the lighter cell present higher fluorescence emission.

Figure S6. Competition between two species, where death rate = 1 for both species and the birth rates are $\lambda_A = 4$ and $\lambda_B = 5$. At t = 0, a square lattice of 50 x 50 sites was filled, and each site was occupied by an A (red) strain or by a B (blue) strain with equal probability of 1/2. The stochastic birth–death process then took place on the lattice (with periodic boundary conditions). As expected, strain B drove strain A into ever-shrinking spatial domains. Panel (a) shows the ratio between A and B strains, panels (b)–(d) are three snapshots of the system at t = 10, t = 100 and t = 200 (generation time).

Figure S7. Relationship between the expression of colicin E2 and GFP encoding genes showing high Pearson correlation coefficient ($r = 0.95$).

Supplementary video legends

Video S1. Colicin E2-expressing cells growing on M9 agar. *E. coli* MG1655 bearing E2pBR322 and E2-pUA66 plasmids was pre-cultured in M9 medium and transferred to an agar pad. Colicin expression in the monolayer sessile culture is represented by green fluorescence.

Video S2. Colicin A-expressing cells growing on M9 agar. *E. coli* MG1655 bearing pBR322-ColA and pUA66-ColA plasmids was pre-cultured in M9 medium and transferred to an agar pad. Colicin expression in the monolayer sessile culture is represented by green fluorescence.

Video S3. Colicin E7-expressing cells growing on M9 agar. *E. coli* MG1655 bearing pBR322-ColE7 and pUA66-ColE7 plasmids was pre-cultured in M9 medium and transferred to an agar pad. Colicin expression in the monolayer sessile culture is represented by green fluorescence.

Video S4. Control cells growing on M9 agar. *E. coli* MG1655 bearing pUA66-ColE2 plasmids was pre-cultured in M9 medium and transferred to an agar pad. Fluorescence expression is monitored in the monolayer sessile culture.

Video S5. Colicin E2-expressing cells growing on M9 agar supplemented with an inducing agent. *E. coli* MG1655 bearing pBR322-ColE2 and pUA66-ColE2 plasmids

was pre-cultured in M9 medium and transferred to an agar pad supplemented with 50 ng mL^{-1} Mitomycin C. Colicin expression in the monolayer sessile culture is represented by green fluorescence.

Video S6. Colicin A-expressing cells growing on M9 agar supplemented with an inducing agent. *E. coli* MG1655 bearing pBR322-ColA and pUA66-ColA plasmids was pre-cultured in M9 medium and transferred to an agar pad supplemented with 50 ng mL^{-1} Mitomycin C. Colicin expression in the monolayer sessile culture is represented by green fluorescence.

Video S7. Colicin E7-expressing cells growing on M9 agar supplemented with an inducing agent. *E. coli* MG1655 bearing pBR322-ColE7 and pUA66-ColE7 plasmids was pre-cultured in M9 medium and transferred to an agar pad supplemented with 50 ng mL^{-1} Mitomycin C. Colicin expression in the monolayer sessile culture is represented by green fluorescence.

Supplementary Tables

Table S1. Statistical values.

Treatment	Colicin type	Statistical test		
		**Test type**		***P*-value**
Cell length	All colicins	One-way ANOVA	f_2=24.02	<0.001
	Colicin A expressers vs. non-expressers cells	Student's t-test	t_{98}=5.91	<0.001
	Colicin E2 expressers vs. non-expressers cells	Student's t-test	t_{98}=10.65	<0.001
	Colicin E7 expressers vs. non-expressers cells	Student's t-test	t_{98}=8.72	<0.001
	Fluorescent expressers vs. non-expressers cells (colicin free)	Student's t-test	t_{98}=-0.10	0.460
	Colicin A expressers vs. non-expressers cells induced by MitC	Student's t-test	t_{98}=4.28	<0.001
	Colicin E2 expressers vs. non-expressers cells induced by MitC	Student's t-test	t_{98}=5.17	<0.001
	Colicin E7 expressers vs. non-expressers cells induced by MitC	Student's t-test	t_{98}=5.57	<0.001
Cell doubling time	All colicins	One-way ANOVA	f_2=0.47	0.620
	Colicin A expressers vs. non-expressers cells	Student's t-test	t_{98}=1.04	0.299
	Colicin E2 expressers vs. non-expressers cells	Student's t-test	t_{98}=1.37	0.172
	Colicin E7 expressers vs. non-expressers cells	Student's t-test	t_{98}=0.34	0.728
	Colicin A expressers vs. non-expressers cells induced by MitC	Student's t-test	t_{98}=5.71	<0.001
	Colicin E2 expressers vs. non-expressers cells induced by MitC	Student's t-test	t_{98}=5.57	<0.001
	Colicin E7 expressers vs. non-expressers cells induced by MitC	Student's t-test	t_{98}=5.10	<0.001

Table S2. The growth rate per minute of the strain used in this study.

	**Strain**	**Subpopulation**	**Growth rate (min^{-1})**
Uninduced	Colicin A	Expressers	0.028 ± 0.008
		Non-expresses	0.034 ± 0.003
	Colicin E2	Expressers	0.026 ± 0.008
		Non-expresses	0.033 ± 0.004
	Colicin E7	Expressers	0.027 ± 0.008
		Non-expresses	0.034 ± 0.003
Induced with Mitomycin C	Colicin A	Expressers	0.015 ± 0.005
		Non-expresses	0.030 ± 0.006
	Colicin E2	Expressers	0.02 ± 0.009
		Non-expresses	0.028 ± 0.006
	Colicin E7	Expressers	0.015 ± 0.004
		Non-expresses	0.028 ± 0.007

Table S3. Bacterial strains and plasmids used in this study.

Bacterial strains/plasmids	Relevant properties	Ref
**Bacterial strains**		
**MG1655**	F^-, λ^- ilvG- rfb-50 rph-1	[3]
**Plasmids**		
**pBR322-ColA**	pBR322 *(caa, cai, cal)*, Amp^R; (colicin A)	[4]
**pBR322-ColE2**	pBR322 (*ce2a*, *ce2i*, *ce2l*), Tet^R, Amp^R; (colicin E2)	[5]
**pBR322-ColE7**	pBR322 *(ce7a*, *ce7i*, *ce7l),* Tet^R, Amp^R; (colicin E7)	[6]
**pUA66**	SC101, GFP*mut2*, Kan^R; (promoterless)	[7]
**pUA66-ColA**	Colicin A promoter::pUA66, Kan^R	This study
**pUA66-ColE2**	Colicin E2 promoter::pUA66, Kan^R	This study
**pUA66-ColE7**	Colicin E7 promoter::pUA66, Kan^R	This study

Table S4. Primers used in this study.

	Target	Forward primer (5'→3')	Reverse primer (5'→3')
**Plasmid construction**	Colicin A promoter	GTGCTCGAGATTTTCCCG	CCACCATAATTGGATCCA
	Colicin E2 promoter	GCTCGAGTGGAAGCGGGAC	CGGATCCGCTCATAAAAATTCC
	Colicin E7 promoter	ACTCGAGAGCGGCGGTGT	CGTCCGGATCCACCGCTC
**qPCR analysis**	Colicin E2	AGTAGGTGGTAGGGAACGCTTTG	TGTCGCTTAGGTGTGGTCACT
	GFP	AGGTGATGCAACATACGGAA	TGATCTGGGTATCTCGCAAA

Figures

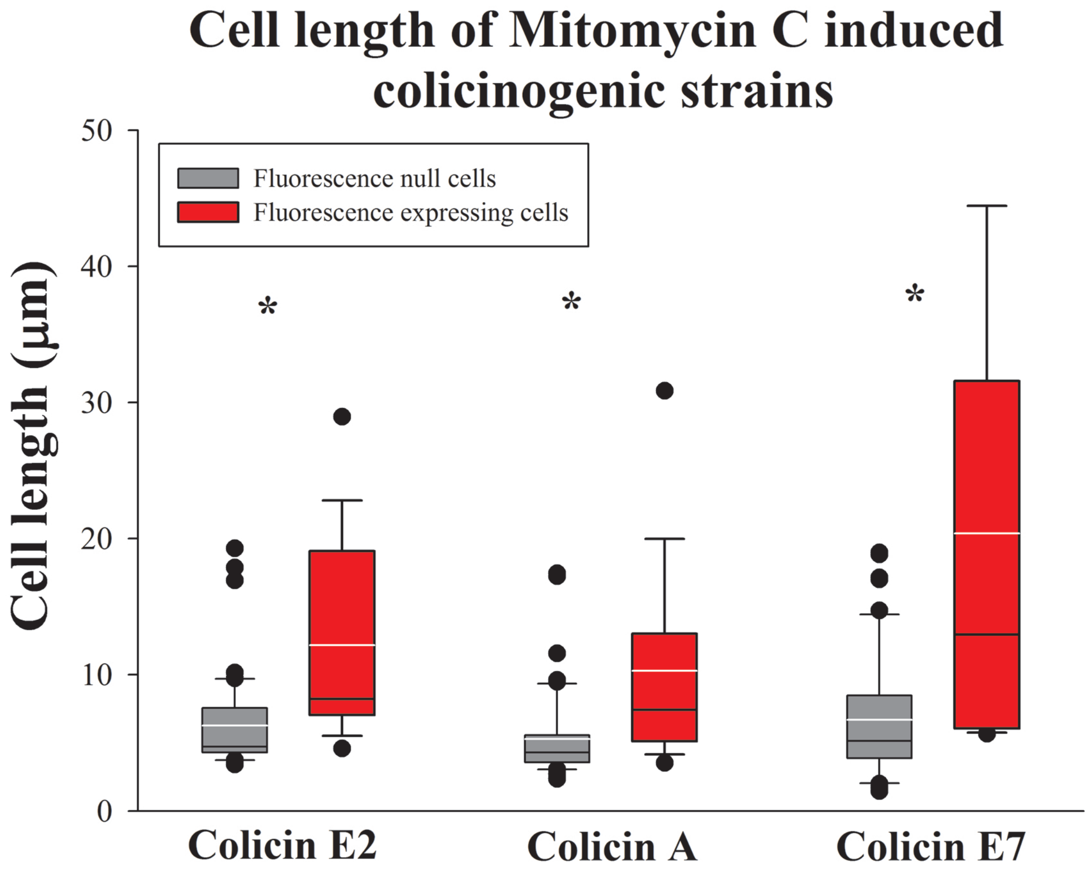

Figure 1. Cell length of colicinogenic strains under induced conditions. The length of colicin expressers (red box) and non-expressers (grey box) was compared for colicins A, E2 and E7 (n = 50) depicted by fluorescence emission. Error bars refer to the 10/90th percentiles of the tested data and whiskers to 5/95th percentile. White line indicates mean and black line indicates median. White line indicates mean and black line indicates median. The asterisk (*) points to significant differences between the length of fluorescent cells and cells that did not fluoresce ($P < 0.001$ by Student's t-test).

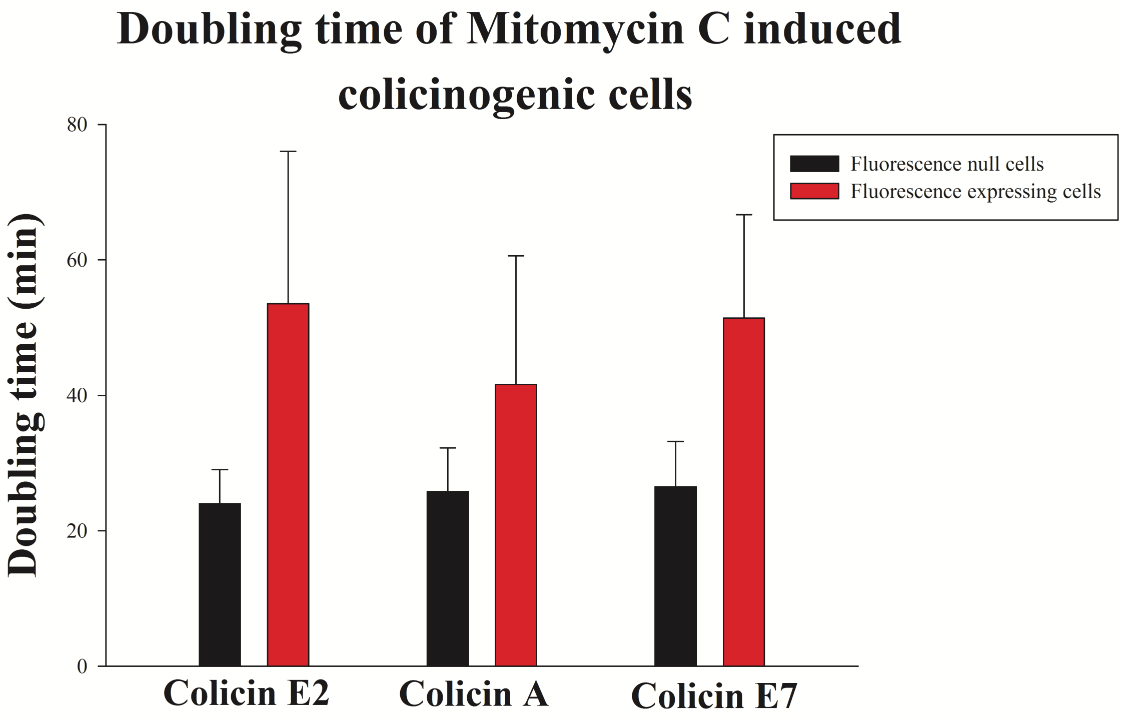

Figure S2. Cell doubling time of colicinogenic strains under induced conditions. Bars represent the average cell doubling time and standard deviation for expressers (red bar) and non-expressers (black bar) of colicins A, E2 and E7 (n = 50 for E2, n = 20 for A, E7). Asterisk (*) indicates significant differences between the doubling time of fluorescent cells and cells that did not emit fluoresce ($P < 0.001$ by Student's t-test).

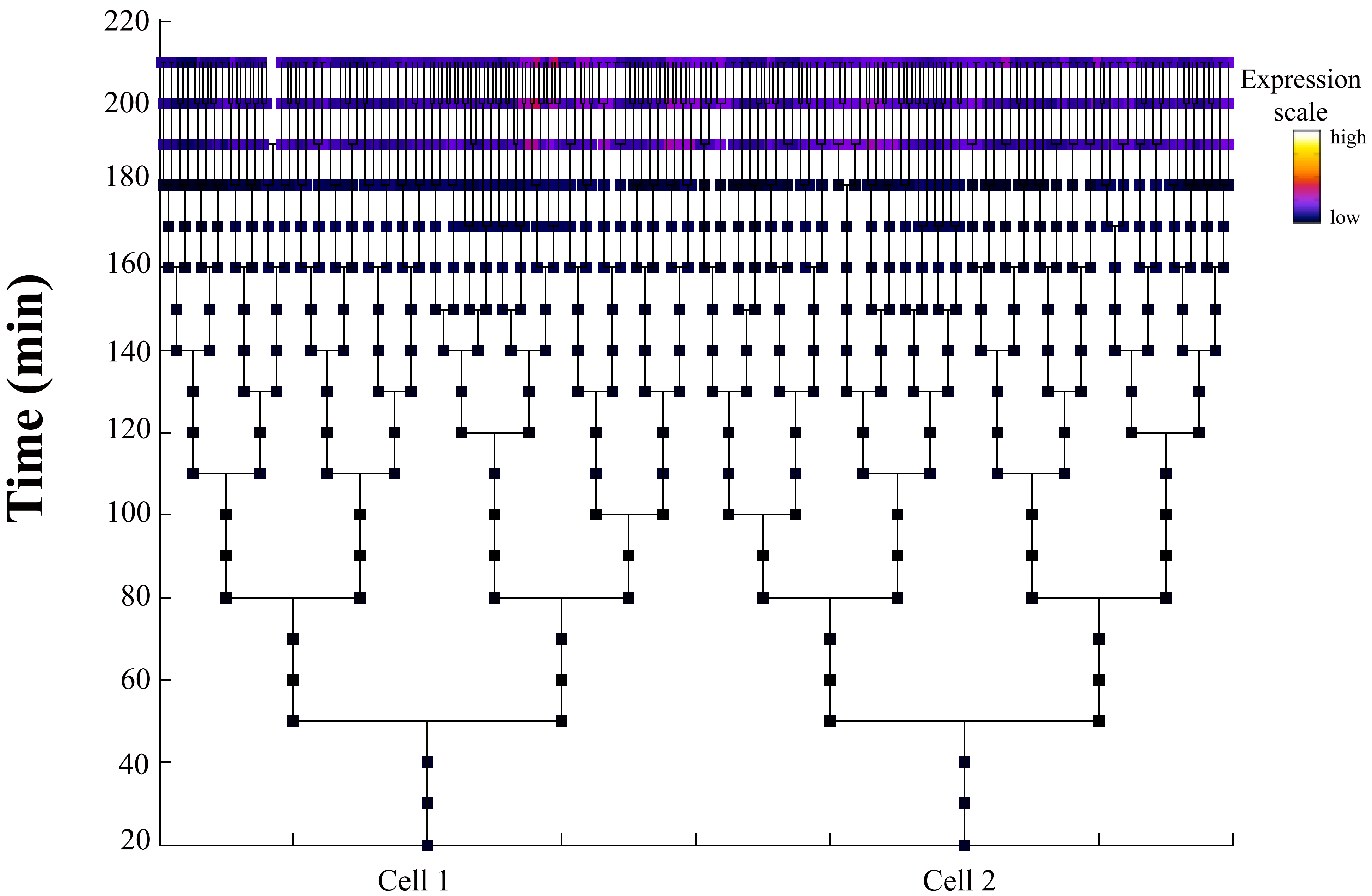

Figure S3. Representative cell lineage of strain expressing colicin A. Colicin expression was monitored over time (y axis) along the lineages of two cells. Expression is depicted by colour: dark cells are non-expressers while lighter cells, due to higher fluorescence emission, mark colicin expression.

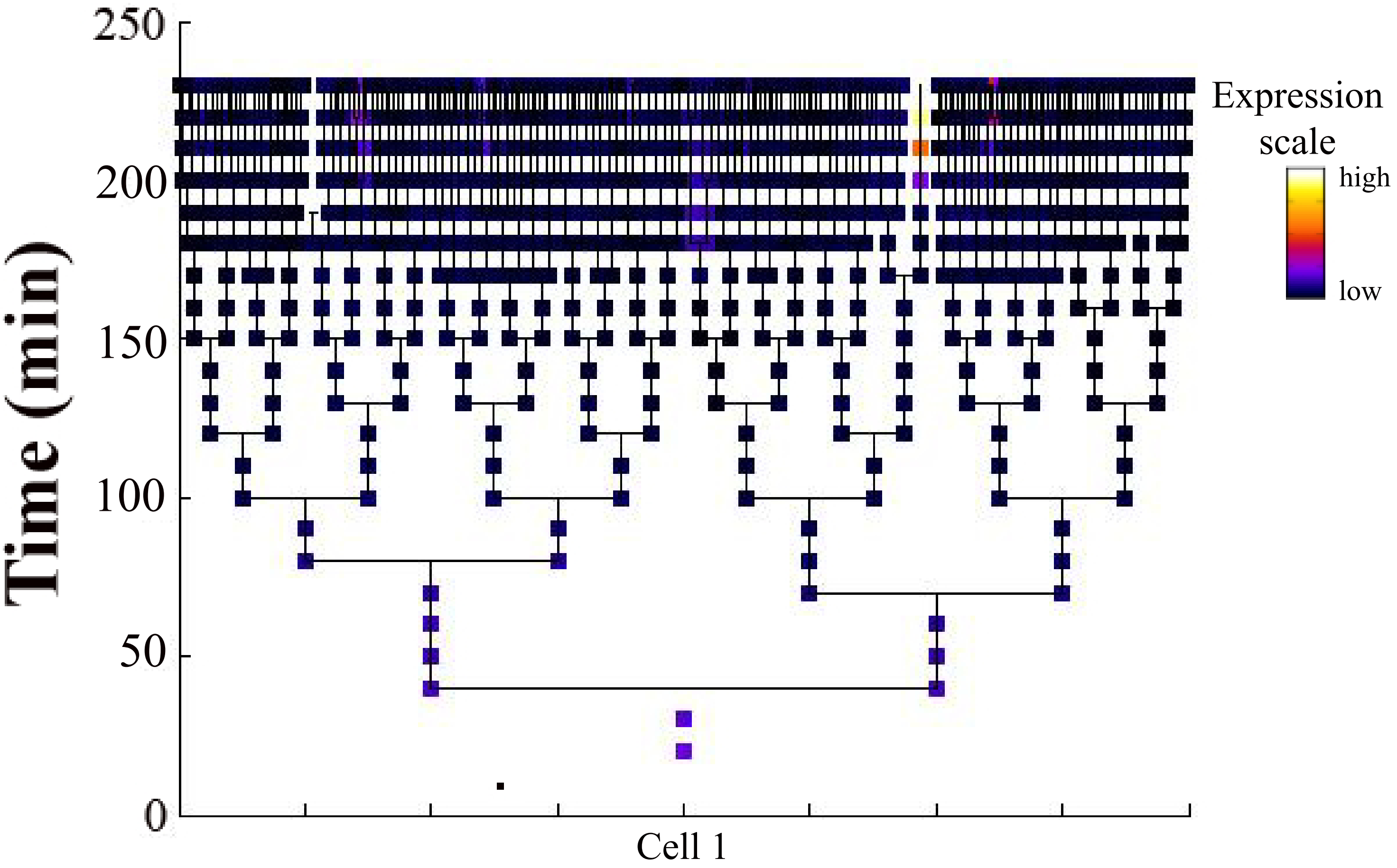

Figure S4. Representative cell lineage of strain expressing colicin E7. Colicin expression was monitored over time (y axis) along the lineages of one cell. Expression is depicted by colour: dark cells are non-expressers while lighter cells, due to higher fluorescence emission, mark colicin expression.

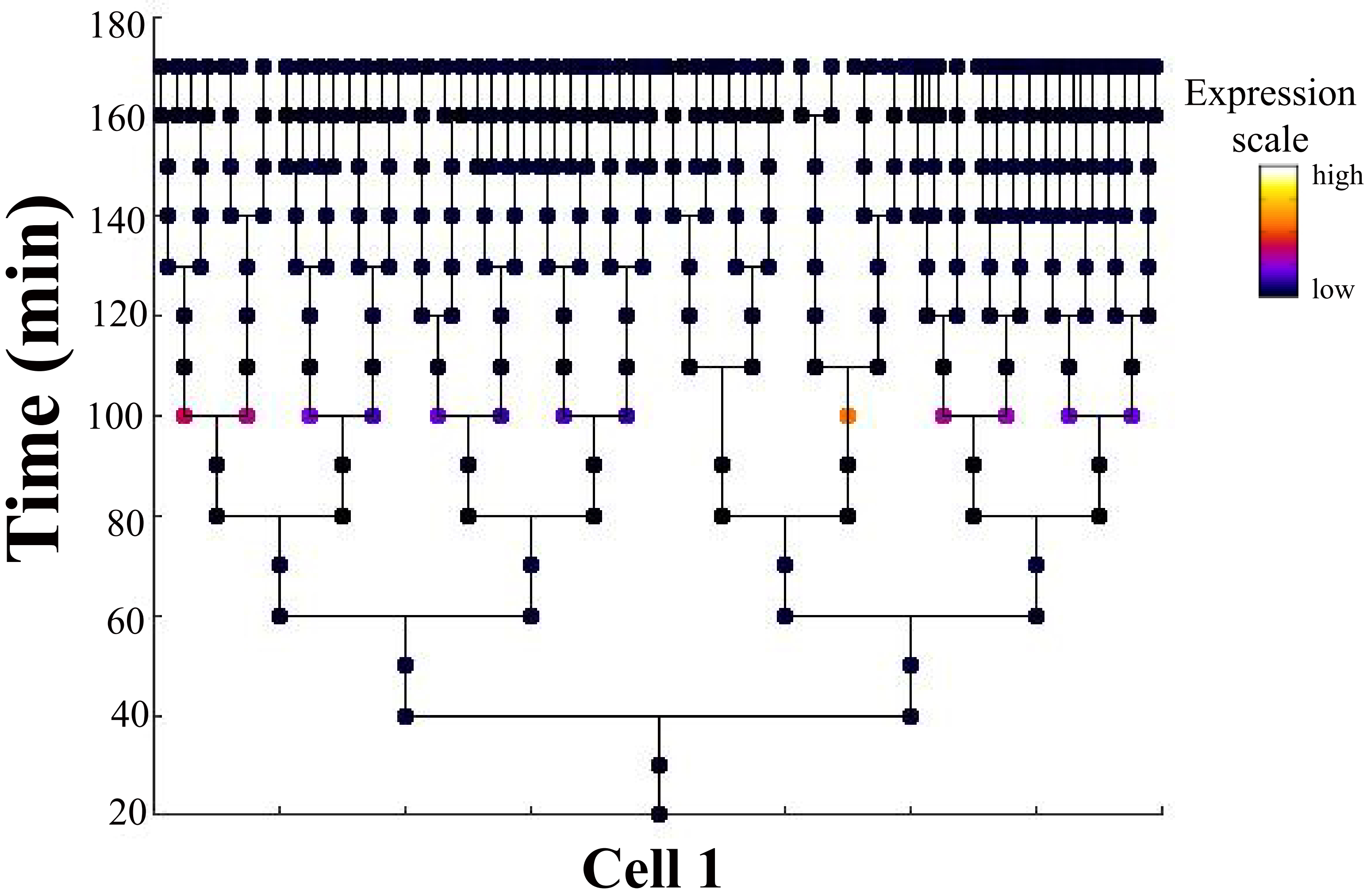

Figure S5. Representative cell lineage of control strain lacking the colicin-encoding genes. GFP expression was monitored over time (y axis) along the lineages of one cell. Expression was depicted by color: dark cells are non-expressers while lighter cells mark expression such that the lighter cell present higher fluorescence emission.

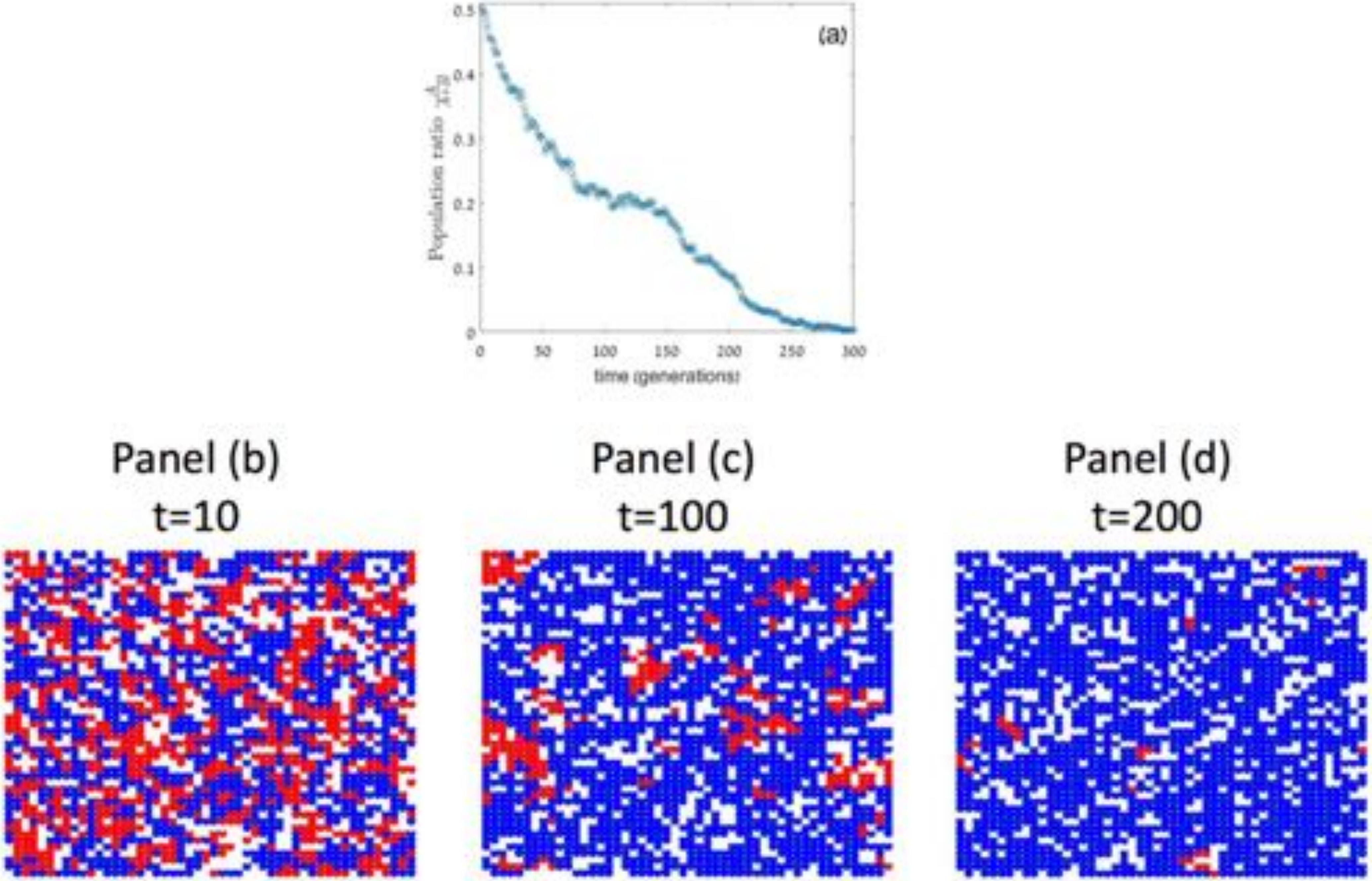

Figure S6. Two species competition, where the death rate is one for both species and the birth rates are λA=4 and λB=5. At t=0 a square lattice of 50X50 sites was filled, each site is occupied by an A (red) strain or by a B (blue) strain with equal probabilities 1/2. The stochastic birth-death process then take place on the lattice (with periodic boundary conditions). As expected, strain B drove strain A into ever shrinking spatial domains. Panel (a) shows the ratio between A and B strains, panels (b)-(e) are three snapshots pf the system at t=10, t=100 and t=200 (generation time).

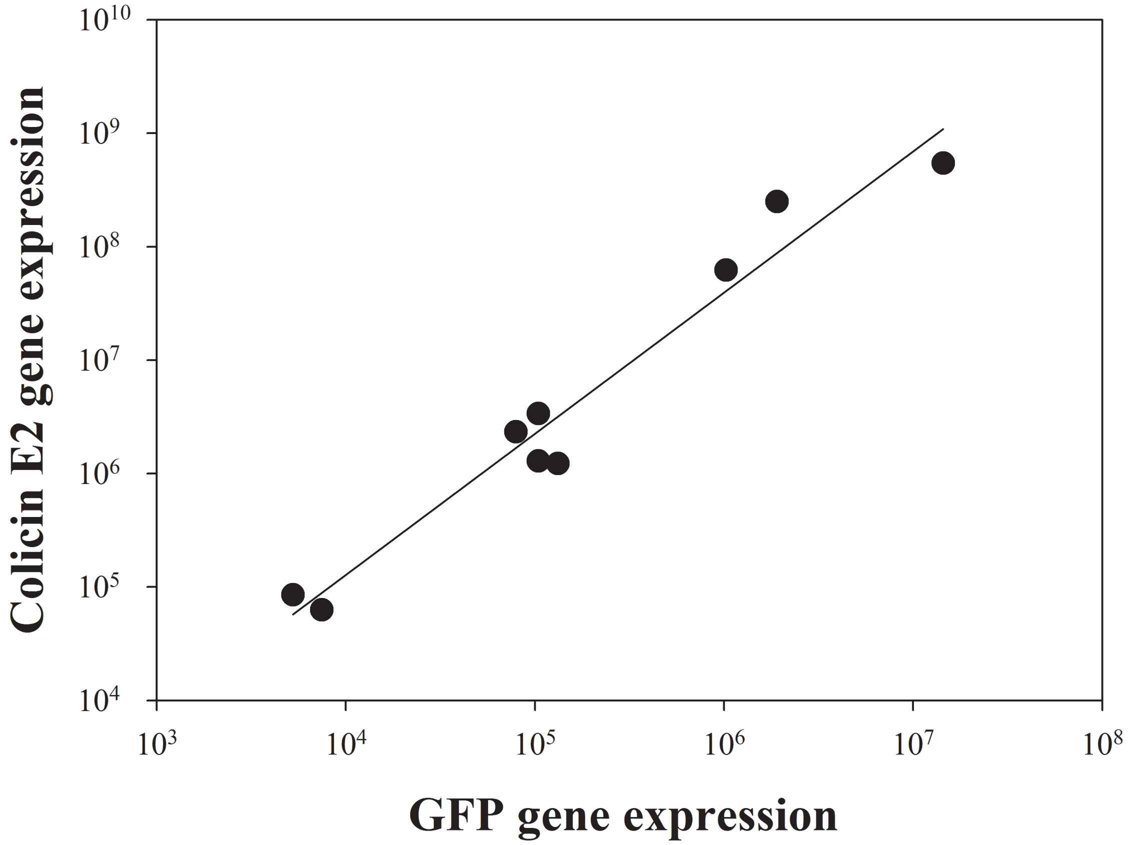

Figure S7. Relationship between the expression of colicin E2 and GFP encoding genes showing high Pearson correlation coefficient (r = 0.95).

References

1. Cascales, E. *et al.* Colicin Biology. *Microbiol Mol Biol Rev* **71,** 158–229 (2007).
2. Kearse, M. *et al.* Geneious Basic: An integrated and extendable desktop software platform for the organization and analysis of sequence data. *Bioinformatics* **28,** 1647–1649 (2012).
3. Guyer, M. ., Reed, R. ., Steitz, J. . & Low, K. B. Identification of a Sex-factor-affinity Site in E. coli as γδ. *Cold Spring Harb. Symp. Quant. Biol.* **45,** 135–140 (1981).
4. Majeed, H., Lampert, A., Ghazaryan, L. & Gillor, O. The Weak Shall Inherit: Bacteriocin-Mediated Interactions in Bacterial Populations. *PLoS ONE* **8,** e63837 (2013).
5. Ghazaryan, L., Soares, M. I. M. & Gillor, O. Auto-regulation of DNA degrading bacteriocins: Molecular and ecological aspects. *Antonie Van Leeuwenhoek Int. J. Gen. Mol. Microbiol.* **105,** 823–834 (2014).
6. Ghazaryan, L., Tonoyan, L., Ashhab, A. Al, Soares, M. I. M. & Gillor, O. The role of stress in colicin regulation. *Arch. Microbiol.* **196,** 753–764 (2014).
7. Zaslaver, A. *et al.* Just-in-time transcription program in metabolic pathways. *Nat. Genet.* **36,** 486–491 (2004).
